# A Linguistic Analysis of Future Perceptions of Parents of Adolescents With Complex Regional Pain Syndrome and Parents of Pain‐Free Peers

**DOI:** 10.1002/ejp.70072

**Published:** 2025-06-27

**Authors:** Amy Parkinson, Line Caes, Emma Fisher, Abigail Jones, Melanie Noel, Abbie Jordan

**Affiliations:** ^1^ Department of Psychology University of Bath Bath UK; ^2^ Division of Psychology, Faculty of Natural Sciences University of Stirling Stirling UK; ^3^ Department of Health & Centre for Pain Research University of Bath Bath UK; ^4^ College of Health, Science, and Society University of the West of England Bristol UK; ^5^ Department of Psychology University of Calgary Calgary Alberta Canada; ^6^ Alberta Children's Hospital Research Institute Calgary Alberta Canada; ^7^ Department of Psychology & Centre for Pain Research University of Bath Bath UK

## Abstract

**Background:**

Complex regional pain syndrome (CRPS) is a chronic pain condition which typically affects a body extremity and occurs more frequently from the onset of adolescence. Parents of adolescents with chronic pain report uncertainty and fear concerning their adolescent's transition to adulthood. Nonetheless, little is known about how parents of adolescents with CRPS perceive their adolescent's future.

**Methods:**

This study sought to explore the future perceptions of parents of adolescents with CRPS (*n* = 45) and compare their use of language with a sample of parents of adolescents without chronic pain (*n* = 50). All participants completed an online story completion task in which they described their imagined future for their adolescent (14–25 years). Responses were coded using a linguistic analysis programme and the significance of group differences was tested using inferential statistics.

**Results:**

Parents of adolescents with CRPS used significantly more negative emotion and anger words, and their responses had an overall lower emotional tone score (i.e., were more negative) than parents of pain‐free adolescents. Parents of pain‐free adolescents used significantly more certainty and friends words. Adolescent age and gender predicted a significant amount of variance in use of family words. No group differences were found for the remaining dimensions.

**Conclusions:**

Key differences exist regarding expectations of their adolescent's future between parents of adolescents with and without chronic pain. Given the important contribution of parents to the pain experience of their adolescent, and the influence of parental expectations on future outcomes, interventions should target the future‐focused expectations of parents.

**Significance Statement:**

Parental expectations of their adolescent's future may be impacted by the presence of their adolescent's chronic pain condition. Our findings point to the importance of considering and targeting parental future perceptions in paediatric chronic pain interventions.

## 1 Introduction

Complex regional pain syndrome (CRPS) is a chronic pain condition which typically affects bodily extremities (Weissmann and Uziel 2016). In children and adolescents, CRPS has been associated with higher levels of pain intensity and functional disability compared with other pain conditions (Logan et al. 2013). Peak onset of paediatric CRPS occurs during adolescence (Borucki and Greco 2015), a distinct developmental period in which goal setting and future planning become increasingly important (Beal and Crockett 2010). Research demonstrates that living with chronic pain shapes adolescents' perceptions about their future (Jones et al. 2020).

The Linguistic Inquiry and Word Count (LIWC; Pennebaker, Booth, et al. 2015; Pennebaker, Boyd, et al. 2015) is a computerised text analysis programme which codes texts on a word‐by‐word basis and determines the total percentage of words within predetermined word categories. Using the LIWC, Nimbley et al. (2021) found that adolescents with CRPS used significantly fewer words pertaining to positive affect and significantly more words pertaining to anger and negative affect than adolescents without chronic pain when describing their future (Nimbley et al. 2021).

The impacts of adolescent chronic pain are extensive, with parents reporting challenges associated with supporting their child to manage their pain (Cox et al. 2022 ; Jordan et al. 2007). Parents of children with long‐term conditions seek information from parents of children living with similar conditions. Such social comparisons seemingly influence perception of their child's situation (Hodges and Dibb 2010). Research indicates that greater parental social comparison is associated with poorer child outcomes (Mendes et al. 2017).

Qualitative research has shown that parents of adolescents with chronic pain experience uncertainty and distress concerning their child's transition to adulthood (Le et al. 2019). Normative research has determined that parental expectations of, and aspirations for, their adolescent's future are associated with adolescent future outcomes (Pinquart and Ebeling 2020; Irwin and Elley 2013). No study has yet examined differences in future perceptions between parents of adolescents with and without chronic pain.

This study builds on Nimbley et al. (2021) by examining how parents of adolescents with CRPS perceive living with CRPS will impact their adolescent's future. We will focus on domains affected by adolescent chronic pain including experiences of ‘positive’ and ‘negative’ emotions, friendships and family relationships, (Cruz et al. 2011; Forgeron et al. 2010). The LIWC cognitive processes subcategories (insight, tentativeness, certainty, causation, differentiation and discrepancy) will be included as we anticipate that the distinct experience of parents of adolescents with CRPS, and how the condition likely influences how often and in what ways parents think about their adolescent's future, will impact both the types and level of cognitive processing undergone during our study task.

It was hypothesised that parents of adolescents with CRPS would use significantly fewer positive affect, friends and certainty words and significantly more negative emotion, anger, sadness, anxiety, family, insight, causation, differentiation, discrepancy and tentativeness words than parents of pain‐free adolescents. It was also hypothesised that responses of parents of adolescents with CRPS would have a significantly lower overall emotional tone score (be more negative), than parents of pain‐free adolescents.

## 2 Methods

This study adopted a between‐subjects, cross‐sectional design to compare the language used by parents of adolescents with CRPS and parents of pain‐free adolescents in a story completion task. We used data collected as part of a larger research project exploring future perceptions in adolescents with CRPS and their parents. The project's full protocol was registered on the Open Science Framework (see https://osf.io/r2wsc/). The larger research project examined how pain and pain memories influence adolescent future identity and parental perceptions during a critical stage of identity development. Within this broader aim, this study used linguistic software to compare parental views on their adolescent's future between CRPS and pain‐free groups. It aligns with Jones et al. (2020) and Nimbley et al. (2021), both of which analysed data from the same dataset. Specifically, Jones qualitatively examined adolescent story completion and interview data, while Nimbley used LIWC software to compare future perceptions between adolescents with CRPS and pain‐free adolescents. This paper adopts a similar approach to Nimbley's but focuses on analysing parental data.

Ethical approval for the larger research project was granted by the Psychology Research Ethics Committee at the University of Bath (PREC 18‐112).

### 2.1 Procedure

Recruitment ran between May 2018 and September 2019. Participants were recruited via advertisements posted on social media platforms (e.g., Facebook and Twitter/X) and at local UK schools and community centres. Parents of adolescents with CRPS were also recruited via advertisements posted in CRPS‐related charity newsletters, blogs and forums (e.g., Reddit).

To be eligible to participate in the study, participants were required to be a parent of an adolescent between the ages of 14 and 25 years (Sawyer et al. 2018). This extended definition of adolescence was adopted to reflect more recent delays in completion of social and autonomy related milestones (e.g., becoming financially independent) in addition to structural brain changes that occur into the mid‐twenties (Sawyer et al. 2018; Jones et al. 2023). Fourteen years of age was allocated as the lowest eligible age as data for this study were collected concurrently with the collection of adolescent data. Patient and Public Involvement and Engagement prior to starting the study identified that this task was too cognitively demanding for those under the age of 14 years. Additional eligibility criteria required participants to: (a) be fluent in English; (b) have the capacity to give informed consent; (c) have internet access; and (d) have no self‐reported severe mental health condition (e.g., psychosis). An eligibility criterion for the pain‐free group was the self‐report that their adolescent did not experience chronic pain (defined as pain occurring on most days over the past 3 months). For the sample of parents of adolescents with CRPS, participants self‐reported their adolescent's formal diagnosis of CRPS, therefore this could have included type 1 or type 2 CRPS diagnoses.

Data were collected using the online survey platform Qualtrics, (2019, https://www.qualtrics.com). Following issues with fraudulent responses during early stages of recruitment, the recruitment process was amended so that individuals who showed interest in the study were emailed with relevant study information and screened for their eligibility with a series of questions (e.g., where they saw the study advertised) before being sent a direct link to the online survey. Any participants who could not answer these questions were not provided with a link to the survey. Additional measures to ensure data integrity included checking IP addresses for duplication and checking all responses to the story completion task carefully to ensure that they sounded genuine rather than completed by a ‘bot’. Additionally, timing of responses was monitored, with specific attention paid to multiple responses received in short term frames. All ‘suspicious’ responses were then double checked by the senior author before a decision was made concerning inclusion or exclusion. Responses were only included in the study if both the researcher and the senior author felt that they were genuine.

The survey comprised participant demographic questions which included participants' gender, participants' age, country of inhabitation, highest level of education obtained, employment status, relationship status, relationship to adolescent (e.g., mother), number of children, parents' own experience of chronic pain and parents' current pain intensity (if appropriate). Regarding their adolescent, participants were asked to specify their adolescent's gender, age, length of time since diagnosis (if appropriate), current pain level (if appropriate), whether their adolescent was currently in education or work. Following the demographic section, participants were directed to the story completion task and subsequent study tasks. These final tasks included describing their imagined future of a friend of their child and a pain memory task in which participants were asked to recall and describe a time in which they experienced pain. At the end of the survey, participants were shown a final debrief screen. To thank participants for their time, they were offered the option to receive an online shopping voucher to the value of £10.

### 2.2 Participants

Parents of adolescents who were pain‐free comprised 50 participants whilst the parents of adolescents with CRPS group comprised an initial sample size of 51 participants. Within the parent of adolescents with CRPS group, one response was excluded due to a participant completing the survey twice, and five responses were excluded as suspected fraudulent responses, leaving a final sample size of 45 participants for the CRPS parent group. Details of participants in both groups can be found in Table 1.

**TABLE 1:** Participant demographic information.

	Parents of adolescents with CRPS (*n* = 45)	Parents of adolescents who are pain‐free (*n* = 50)
Age (years)	*M* = 47.09, SD = 6.08	*M* = 48.82, SD = 6.82
Gender (%)		
Female	91% (*n* = 41)	86% (*n* = 43)
Male	9% (*n* = 4)	14% (*n* = 7)
Adolescent age (years)	*M* = 17.40, SD = 3.04	*M* = 19.86, SD = 3.45
Adolescent gender (%)		
Female	73% (*n* = 33)	44% (*n* = 22)
Male	27% (*n* = 12)	56% (*n* = 28)

### 2.3 Story Completion

The story completion method (Clarke et al. 2019) is a novel qualitative method in which participants are provided with a story ‘stem’ and invited to complete the story using free text. This method enables and encourages a breadth of opinions toward sensitive topics like sex offending (Gavin 2005) and orgasmic absence (Frith 2013), which may be harder to explore using more traditional qualitative means of data collection (e.g., interviews, focus groups; Clarke et al. 2019). Whilst sample sizes in story completion research vary substantially, studies typically recruit between 40 and 60 participants (Jones et al. 2020; Clarke et al. 2019).

As this study aimed to explore parents' expectations for their adolescent's transition into adulthood, a third‐person future perspective story stem was chosen with the setting of a secondary high school reunion, an event considered universally applicable and meaningful. Similar to the story stem for adolescents in the Nimbley et al. (2021) paper, this story stem used the same words yet asked parents about their child's imagined future rather than asking adolescents about their imagined future. The story stem for the present study was: ‘Imagine that it has been 10 years since your child has graduated from secondary school. They are at their high school reunion and hearing what their friends have been doing since they finished school. What will your child's story look like?’. To avoid directing parents of adolescents with CRPS to an illness narrative, an identical story stem was used for both participant groups. Participants were encouraged to write in as much detail as possible so that sufficient data were collected for analysis, with a minimum length of 900 characters set. The range in story length was 153–444 words. The mean story length was 213 words.

### 2.4 Linguistic Coding

Participant responses were coded using LIWC2015 (Pennebaker, Booth, et al. 2015; Pennebaker, Boyd, et al. 2015). LIWC is a computerised text analysis tool which codes text and calculates the percentage of words that fall into over 60 linguistic dimensions and word categories, as well as providing other output variables (e.g., emotional tone). Each LIWC word subcategory comprises a dictionary of words, developed and refined by human judges using a robust process outlined by Pennebaker, Booth, et al. (2015), Pennebaker, Boyd, et al. (2015). Since release of the first versions of LIWC (Francis 1993; Pennebaker et al. 2001), studies have found the software valid, reliable and superior to other computerised text analysis tools (Alpers et al. 2005; Pennebaker and Francis 1996; Bantum and Owen 2009).

Research using the LIWC illustrates the human tendency to use certain types of word in different situations. For instance, descriptions of happy events typically include many positive emotion words and few negative emotion words (Kahn et al. 2007). Subcategories of cognitive words like causal and insight words are likely to be present in narratives of challenging situations or experiences (Klein and Boals 2010), and uncertainty about a topic tends to induce the use of tentative language, with the reverse being true for topics about which the speaker has certainty (Pasupathi 2007). For a comprehensive review of the various word categories included in the LIWC, see Tausczik and Pennebaker (2010). Thus, coding texts based on the various LIWC dimensions can reveal valuable information about a speaker. Previous studies have used LIWC to explore factors within chronic pain including pain acceptance (Kim et al. 2021), pain catastrophising (Langer et al. 2016), and, in adolescents, future perceptions (Nimbley et al. 2021).

Prior to coding, all responses were anonymised, all pertinent spelling mistakes corrected, and all duplicate words removed. As the LIWC software codes and categorises words and word stems via their spelling, it was important to correct obviously misspelled words to ensure that all relevant words were included in appropriate coding categories (Pennebaker, Booth, et al. 2015; Pennebaker, Boyd, et al. 2015). A total of 41 words were altered/removed (if repeated); 0.25% total CRPS parent responses, 0.18% total pain‐free parent responses; for example ‘sufffered’ was altered to ‘suffered’ and ‘struggeling’ was altered to ‘struggling’.

Responses were coded according to 14 of the LIWC's dimensions (see Table 2) falling under the broader categories of affective, social and cognitive words. LIWC's ascribed emotional tone score was also of interest. Emotional tone and the affective and social categories were selected for inclusion as the literature indicates a clear impact of CRPS, and chronic pain more broadly, within psychological (Soltani et al. 2019) and social (Wakefield et al. 2018) domains. The cognitive processes categories were selected for inclusion as it was anticipated that parents of adolescents with CRPS may undergo different cognitive processing during consideration of their child's future as a result of challenging past experiences related to their child's pain condition.

**TABLE 2:** LIWC categories and subcategories relevant to the current study.

Category	Definition	Examples
**Emotional tone**	The proportion of positive emotion vs. negative emotion words	
**Affective processes**	*Words with an emotional content*	Happy, cried and proud
Positive emotion	Words expressing positive emotion	Love and good
Negative emotion	Words expressing negative emotion	Hurt, pain and prejudice
Anxiety	Words expressing feelings of anxiety	Worried and fearful
Anger	Words expressing feelings of anger	Hate, fight and annoyed
Sadness	Words expressing feelings of sadness	Crying, grief and sad
**Social processes**	*Words pertaining to friends, family*	Mate, talk and child
Family	Words pertaining to family	Daughter and dad
Friends	Words pertaining to friendships	Friends and buddy
**Cognitive processes**	*Words pertaining to thoughts or beliefs*	Cause, know and ought
Insight	Words pertaining to reflection	Learned, acceptance
Causation	Words pertaining to causal relationships	Because and effect
Discrepancy	Words related to differences, lack of similarities	Should and couldn't
Tentative	Words pertaining to hesitation	Maybe and perhaps
Certainty	Words pertaining to conviction, assuredness	Always and never
Differentiation	Words pertaining to distinguishing between things	Hasn't, but and actually

*Note:* Adapted from Nimbley et al. (2021).

Within the affective processes category, words pertaining to positive emotion, negative emotion, anxiety, anger and sadness were coded. Within the social category, words pertaining to friends and family were coded. Lastly, within the cognitive category, words indicative of insight, causation, discrepancy, tentativeness, certainty and differentiation were coded.

To address the potential influence of negations (e.g., *not*, *without*) on LIWC word counts, the authors analysed a random sample of 20 stories: 10 from parents of adolescents with CRPS and 10 from parents of pain free adolescents. All instances of negation within these narratives were identified, and a comprehensive list is provided in File S1. Excluding the instance of ‘may or may not’, where the negations balanced each other out, a total of 6 negations were identified across the 20 stories. This suggests that negation was not a significant confounding factor in this study.

### 2.5 Data Analysis

Following the coding of the story completion responses, data analysis was conducted using SPSS. Descriptive analyses (means, standard deviations, minimum and maximum) were conducted for each dependent variable. An independent samples *t*‐test was completed to ascertain whether the adolescent populations differed significantly in terms of age, and a chi‐square explored gender differences across both adolescent and parent groups. Next, hierarchical linear regressions were performed for each dependent variable. Adolescent age and gender were entered into the first step, and group was entered into the second step, allowing us to identify the distinct influence of group membership on use of the word categories and on emotional tone score. Variables of adolescent gender and adolescent age (whom the parents were writing their story about) were included based on literature indicating these factors are associated with the prevalence (Huguet and Miró 2008) and impact of adolescent chronic pain on both the adolescent themselves (Nilsson et al. 2009) and their parents (Eccleston et al. 2004).

## 3 Results

Among the parental groups (adolescent CRPS versus pain‐free adolescents), there were significant differences in terms of the ages and gender of the adolescents whom the parents were writing about. Overall, adolescents without chronic pain were older (mean age 19.96 years vs. 17.40 years) and more likely to identify as male (56% vs. 27%) than adolescents with CRPS. Further, parents of adolescents with CRPS were more likely to be mothers than parents of adolescents without CRPS (91% vs. 86%).

An independent samples t‐test was completed to determine whether there was a significant age difference between adolescents with CRPS (17.40, SD = 3.04) and adolescents without chronic pain (*M* = 19.86, SD = 3.45). Levene's test for equality of variances indicated that the assumption of homogeneity of variance was met, *F* (1, 93) = 1.109, *p* = 0.295. Findings showed that adolescents without chronic pain were significantly older than adolescents with CRPS, *t*(93) = −3.558, *p* = 0.001.

A chi‐square test was conducted to explore gender differences across the adolescent populations. Findings showed that there was a significant relationship between gender and group membership (*χ*(1, *N* = 95) = 8.36, *p* = 0.004). There were more females than males in the CRPS group (33 vs. 12) whereas the pain‐free group had a more balanced distribution (28 males vs. 22 females).

A chi‐square test was conducted to examine the relationship between parent gender and group membership. The results indicated that there was no association between parent gender and group membership (*χ*(1, *N* = 95) = 0.54, *p* = 0.462).

Independent samples *t*‐tests were completed to ascertain whether groups differed significantly in their use of the different word categories. *t*‐Tests identified that the parent groups used significantly different numbers of the following word categories: emotional tone, negative emotion, anxiety, anger, family, friends and certainty words. For overall the word categories emotional tone, negative emotion, anger and certainty the assumption of homogeneity of variance was not met (*F*(1, 93) = 6.67–36.75, all *p*'s < 0.05), while for anxiety, family and friends word categories the assumption of homogeneity of variance was met (*F*(1, 93) =0.17–3.191, all *p*'s > 0.05). Responses of parents of adolescents in the pain‐free group had a significantly higher prevalence of emotional tone (*t*(83.382) = −3.098, *p* = 0.001, *d* = −0.645, 95% CI [−1.056, −0.230]), family words (*t*(93) = −2.276, *p* = 0.025, *d* = −0.468, 95% CI [−0.875, −0.058]), and friends words (*t*(93) = −2.093, *p* = 0.20, *d* = −0.430, 95% CI [−0.836, −0.022]) than those of parents of adolescents with CRPS. On the other hand, parents of adolescents with CRPS used significantly more negative emotion words (*t*(64.913) = 5.615, *p* = 0.000, *d* = 1.189, 95% CI [0.749, 1.624]), anxiety (*t*(93) = 1.693, *p* = 0.047, *d* = 0.348, 95% CI [−0.059, 0.753]), anger (*t*(48.966) = 2.939, *p* = 0.003, *d* = 0.633, 95% CI [0.218, 1.044]) and certainty words (*t*(68.377) = 2.642, *p* = 0.001, *d* = 0.558, 95% CI [0.146, 0.967]) than parents of pain free adolescence. Anger words than parents of adolescents without pain. No significant group differences were found for the remaining word categories (positive emotion, sadness, insight, causation, discrepancy and tentative). A summary of descriptive statistics is provided in Table 3.

**TABLE 3:** Descriptive statistics (average % of words, standard deviation, minimum and maximum) of differences between LIWC categories and subcategories for parents of adolescents with CRPS and parents of pain‐free adolescents.

Category/subcategory	Average %	SD	Minimum	Maximum
Emotional tone				
CRPS	62.19**	30.20	5.11	99.00
Pain free	79.59**	23.73	12.51	99.00
Total	71.35	28.22		
Positive emotion				
CRPS	4.21	1.68	1.12	8.82
Pain free	4.50	2.13	0.56	10.14
Total	4.36	1.92		
Negative emotion				
CRPS	1.95**	1.37	0.00	4.97
Pain free	0.67**	0.72	0.00	2.50
Total	1.27	1.25		
Anxiety				
CRPS	0.39*	0.53	0.00	2.46
Pain free	0.23*	0.41	0.00	1.85
Total	0.31	0.48		
Anger				
CRPS	0.25**	0.48	0.00	2.20
Pain free	0.03**	0.12	0.00	0.51
Total	0.13	0.36		
Sadness				
CRPS	0.27	0.42	0.00	1.57
Pain free	0.18	0.35	0.00	1.65
Total	0.22	0.38		
Family				
CRPS	0.70*	0.82	0.00	3.53
Pain free	1.26*	1.43	0.00	8.47
Total	0.99	1.21		
Friends				
CRPS	0.66*	0.65	0.00	2.70
Pain free	0.98*	0.78	0.00	3.70
Total	0.83	0.74		
Insight				
CRPS	2.21	1.37	0.00	6.11
Pain free	1.77	1.37	0.00	5.08
Total	1.98	1.38		
Causation				
CRPS	1.42	1.02	0.00	3.92
Pain free	1.10	0.95	0.00	3.81
Total	1.25	0.99		
Discrepancy				
CRPS	1.58	1.25	0.00	5.14
Pain free	1.44	1.44	0.00	5.56
Total	1.51	1.35		
Tentative				
CRPS	2.43	1.59	0.50	6.33
Pain free	2.86	2.16	0.00	8.64
Total	2.68	1.91		
Certainty				
CRPS	1.43**	1.14	0.00	5.15
Pain free	0.92**	0.65	0.00	2.86
Total	1.16	0.95		
Differentiation				
CRPS	3.08	1.33	0.89	6.08
Pain free	2.84	1.83	0.00	7.14
Total	2.95	1.61		

*Note:* *p < 0.05 and **p < 0.01 indicate statistically significant differences in word use between the two participant groups.

Hierarchical linear regressions were conducted to explore whether group can explain a significant amount of variance in use of our selected word categories. As some tests violated homoscedasticity assumptions, all tests were bootstrapped (1000 samples). The variance inflation factor was 1.24, suggesting there were no issues of multicollinearity.

The combined variables of adolescent age and gender explained a significant amount of variance in use of family words only (*F*(2, 92) = 3.24, *R* ^2^ change = 0.07, *p =* 0.044). Closer inspection revealed that adolescent age significantly predicted use of family words (*B* = 0.083, SE = 0.035, *β* = 0.240, *t*(92) = 2.37, *p* = 0.020), that is, higher adolescent age was associated with higher use of family words. Adolescent gender did not significantly contribute to the model.

**TABLE 4:** Results of hierarchical linear regression analyses of parent group and LIWC dimensions.

	Step		*b*	95% *CI*	*R* ^2^ change	Adjusted *R* ^2^
Emotional tone	1	Adolescent age Adolescent gender	−1.92* 10.49	−3.42, −0.54 −0.74, 22.25	0.01**	−0.01
	2	Group	25.11**	13.70, 35.18	0.16	0.15**
Positive emotion	1	Adolescent age Adolescent gender	−0.07 0.53	−0.17, 0.42 −0.29, 1.42	0.02	−0.01
	2	Group	0.61	−0.21, 1.39	0.02	0
Negative emotion	1	Adolescent age Adolescent gender	0.02 −0.23	−0.04, 0.08 −0.72, 0.26	0.02	0
	2	Group	−1.4**	−1.87, −0.94	0.26**	0.25**
Anxiety	1	Adolescent age Adolescent gender	0.01 −0.01	−0.02, 0.04 −0.21, 0.19	0	−0.02
	2	Group	−0.18	−0.36, −0.01	0.03	0
Anger	1	Adolescent age Adolescent gender	0 −0.03	−0.02, 0.02 −0.19, 0.11	0.10	−0.01
	2	Group	−0.23*	−0.4, −0.09	0.09*	0.07*
Sadness	1	Adolescent age Adolescent gender	0 −0.04	−0.02, 0.02 −0.2, 0.12	0.01	−0.02
	2	Group	−0.9	−0.24, 0.05	0.01	−0.02
Family	1	Adolescent age Adolescent gender	0.07 −0.08	−0.01, 0.13 −0.63, 0.39	0.07*	0.05*
	2	Group	0.37	−0.09, 0.90	0.02	0.06
Friends	1	Adolescent age Adolescent gender	0.01 0.41	−0.03, 0.05 0.09, 0.70	0.06	0.04
	2	Group	0.41*	0.07, 0.73	0.06*	0.09*
Insight	1	Adolescent age Adolescent gender	0.01 0	−0.07, 0.09 −0.50, 0.55	0	−0.02
	2	Group	−0.48	−1.01, 0.10	0.02	−0.01
Causation	1	Adolescent age Adolescent gender	0.01 −0.09	−0.05, 0.08 −0.53, 0.36	0	−0.02
	2	Group	−0.38	−0.89, 0.13	0.03	0
Discrepancy	1	Adolescent age Adolescent gender	0.01 0.22	−0.07, 0.07 −0.32, 0.80	0.01	−0.01
	2	Group	−0.10	−0.69, 0.51	0	−0.02
Tentative	1	Adolescent age Adolescent gender	−0.07 0.49	−0.19, 0.05 −0.33, 1.32	0.01	−0.01
	2	Group	0.74	−0.10, 1.61	0.03	0.01
Certainty	1	Adolescent age Adolescent gender	−0.01 −0.07	−0.06, 0.05 −0.51, 0.37	0.02	0
	2	Group	−0.51*	−0.97, −0.07	0.06*	0.05*
Differentiation	1	Adolescent age Adolescent gender	0.02 0	−0.08, 0.12 −0.71, 0.73	0	−0.02
	2	Group	−0.29	−0.89, 0.35	0.01	−0.03

*Note:* b and CIs from the last step in the analyses are displayed.

* *p* < 0.05.

** *p* < 0.01.

With regard to affective dimensions, there was no significant difference for use of positive emotion, anxiety, or sadness words between the groups. However, significant differences were found for use of negative emotion (*F*(3, 91) = 11.52, *R* ^2^ change = 0.26, *p* < 0.001) and anger (*F*(3, 91) = 3.18, *R* ^2^ change = 0.09, *p* = 0.028) words, with parents of adolescents with CRPS using more of both word categories. Group membership was also significantly associated with emotional tone score (*F*(3, 91) = 6.38, *R* ^2^ change = 0.16, *p* < 0.001). The average emotional tone score of parents in the pain‐free group was higher than that of parents in the CRPS group, indicating that parents in the pain‐free group used more words indicative of positive emotion compared with use of words indicative of negative emotion.

With regard to social dimensions, significant differences were found for use of friends words (*F*(3, 91) = 4.02, *R* ^2^ change = 0.06, *p* = 0.01), with parents of pain‐free adolescents using more words pertaining to friends. No significant group differences were found for use of words pertaining to family.

With regard to cognitive dimensions, parents of adolescents with CRPS were found to use significantly more words pertaining to certainty than parents of adolescents in the pain free group (*F*(3, 91) = (2.49), *R* ^2^ change = 0.06, *p* = 0.19). No significant group differences were found for any of the other cognitive dimensions (insight, causation, discrepancy, tentative and differentiation) (Table 4).

## 4 Discussion

Using linguistic software, our study explored differences in language used by parents of adolescents with CRPS and parents of adolescents who were pain free when describing their adolescent's future. Broadly, consistent with our hypotheses, future‐focused responses of parents of adolescents with CRPS included a higher proportion of negative emotion and anger words and had a lower emotional tone score (i.e., were more negative) than parents of adolescents who were pain‐free. Parents of adolescents with CRPS also used fewer friends words in their description of their adolescent's future, although in contrast to our hypothesis, no significant differences were found in use of family words. Also inconsistent with our hypothesis was our finding that parents of adolescents with CRPS used significantly more words pertaining to certainty than parents of adolescents without pain. No significant group differences were observed for the remaining cognitive processes subcategories.

These findings provide novel understandings of parental perceptions concerning their adolescent's transition to adulthood in the context of CRPS. To our knowledge, this is the first study to use the LIWC with a participant sample of parents of adolescents with CRPS, or chronic pain more broadly, and also the first study to compare future perceptions of parents of adolescents with and without chronic pain. This research builds on the findings of our earlier study (Nimbley et al. 2021) which explored adolescent future perceptions in the context of CRPS, by contributing to our understanding of how parents specifically think about their adolescent's future in the context of this pain condition.

Congruent with Nimbley et al.'s (2021) findings that adolescents with CRPS used significantly more negative emotion and anger words than adolescents without CRPS, we identified identical findings for parents of adolescents with CRPS. Use of words reflecting positive emotion did not significantly differ between groups (CRPS versus pain‐free), indicating that parents had similar expectations of positive experiences or emotions within their child's future. Consequently, the significant difference found in overall emotional tone between groups in this study is driven by the difference in use of negative emotion words. Together these findings indicate that parents of adolescents with CRPS anticipated similar levels of positive experiences, but greater challenges than parents of pain‐free adolescents. Our findings suggest that some individuals maintain a high quality of life despite chronic pain (Parsons et al. 2022). This finding may enhance our understanding of how parents perceive their adolescent's future with CRPS. Prior research links hopeful future expectations in adolescents with chronic pain to better outcomes (Schmid and Lopez 2011; McDade et al. 2011), but the role of parental expectations and their impact on adolescent outcomes remains unclear.

Despite evidence of heightened anxiety in parents of children with chronic pain (Campo et al. 2007), we found no difference in parental use of anxiety words between CRPS and pain‐free groups. This may be because adolescence itself is a universally stressful period for parents (Koning et al. 2013), leading to similar anxiety levels across groups.

Aligning with the findings of Nimbley et al. (2021) but contrary to our hypothesis, we found no significant group differences in family word use. Together with our finding of no significant group differences in the use of anxiety words, these findings could reflect optimism on behalf of parents of adolescents with CRPS that they will recover from their pain condition and lead a ‘normal’ life.

Parents of adolescents with CRPS used fewer friend‐related words than parents of pain‐free adolescents, aligning with our prediction and supporting evidence of peer difficulties in adolescents with chronic pain (Van Alboom et al. 2022; Forgeron et al. 2013). However, this finding is incongruent with Nimbley et al. (2021) work which found no significant difference in the number of friends words used between adolescents with CRPS and adolescents who were pain‐free.

Differences between parental and adolescent perspectives have also been identified in other studies, with Peeters et al. (2014) identifying more positive future perceptions of adolescents with long term conditions compared with parent perceptions in some social contexts. Peeters et al. (2014) describe this difference as the ‘proxy problem’, referencing the discrepancy that can exist between young people's self‐reported health‐related quality of life (HRQOL), and parent‐proxy reports. Combined with our findings, parents may overestimate the negative current and future impacts of chronic pain on their children (Vetter et al. 2012).

Our finding of no differences in the use of insight, causation, discrepancy, tentative, or differentiation words does not support our hypotheses and partially contradicts Nimbley et al. (2021), who found that adolescents with CRPS used more insight and discrepancy words when describing their imagined future than those without chronic pain. Overall, these findings indicate that the parent groups engaged in similar levels of cognitive processing, suggesting that parents may exhibit similar consideration of, or worrying about, their children's future, regardless of circumstances. However, our finding that parents of adolescents with CRPS used more words pertaining to certainty contradicts our hypothesis, and literature reporting high levels of uncertainty faced by parents of adolescents living with chronic pain (Le et al. 2019). This finding may reflect efforts on behalf of this parent group to resolve or even deflect from any uncertainty they do feel in relation to their adolescent's future. Alternatively, when viewed in conjunction with the difference in use of words pertaining to negative emotions and friends, this finding could indicate a sense of resignation on the part of this parent group. Future research could explore this further, including the potential impact of time since diagnosis.

It is possible that our study task may have masked cognitive processing indicators like pauses and hesitations that are typically picked up in speech. Given research indicates additional challenges in the context of parenting a child with CRPS, further research should continue to explore whether these parents do indeed demonstrate different cognitive processing than parents of adolescents without chronic pain.

The present study does of course have limitations. First, the LIWC analyses texts according to pre‐determined dictionaries, which means that contextual clues are not considered (Pilny et al. 2019). It is therefore not possible to determine the valence of words (e.g., ‘my child will be happy’ vs. ‘my child will *not* be happy’). Future research should consider taking a mixed‐methods approach, maintaining the ability to find objective differences in language whilst being able to explore these differences further qualitatively. Second, numerous factors influence the paediatric chronic pain experience and the future expectations of parents of adolescents. Including such factors in our analysis would have strengthened the conclusions to be drawn from our research. For example, Bujnowska et al. (2019) showed that parental education influenced the amount of future anxiety reported by mothers of children with developmental disabilities. Another likely relevant factor is socioeconomic status, which is both associated with risk of developing chronic pain (Prego‐Domínguez et al. 2021) and parental expectations in the broader adolescent population (O'Donnell et al. 2022).

Additionally, we did not ask participants about either their own ethnicity or the ethnicity of their child. Our recruitment strategy may also be considered a limitation; we recruited parents of adolescents with CRPS via internet forums and CRPS groups. This focus could affect the generalisability of our findings to those not actively engaging with such platforms. Furthermore, parents of adolescents with CRPS answered demographic questions, including one about their adolescent's pain, before the story completion task, potentially priming them to consider pain in their responses. Finally, we did not ask participants to specify whether their child was diagnosed with type 1 or type 2 CRPS, which may affect the generalisability of our findings to those in either population subgroups. Future research could investigate whether parental expectations of adolescents with chronic pain are influenced by the parents' own experiences with chronic pain. Additionally, studies should aim to include adolescent comparison groups that are more closely matched in age than those used in the current study.

The current study provides evidence of differences between the future expectations of parents of adolescents with CRPS and those of pain‐free adolescents. This finding is clinically important as it highlights the need to consider parental expectations as a significant factor in the management of adolescent chronic pain. While existing research has primarily focused on parental immediate cognitive and behavioural responses to pain, this study suggests that addressing parental expectations and aspirations for their adolescent could serve as an effective target for intervention.

Future research should explore how these expectations influence adolescent outcomes such as emotional wellbeing, pain management and quality of life, potentially mediated by parental cognitive and behavioural responses. Longitudinal studies should assess changes in expectations over time and their impact on short‐term outcomes like school attendance, social connectedness and academic performance, which contribute to long‐term wellbeing. By linking parental expectations to specific adolescent outcomes, future research can inform targeted strategies to enhance the wellbeing of adolescents with chronic pain.

## 5 Conclusion

Despite the important role that parents play in their adolescent's pain experience, little research has explored the role of parental expectations of their adolescent's future. This study sought to identify differences in the future‐focused expectations of parents of adolescents with CRPS and parents of pain‐free adolescents. Differences found in terms of participants' use of negative emotion, anger, certainty and friends words, as well as the overall emotional tone of their responses, point to the importance of exploring and targeting parental expectations of their child's transition to adulthood in the context of chronic pain. Future studies could employ a mixed methods approach to explore differences in expectations in more depth.

## Author Contributions

A.P. conducted the analyses and prepared the initial draft of the manuscript. L.C. developed the idea for the research, reviewed the analyses and commented on the manuscript. A.Jones gathered the data and contributed to the writing of the paper. E.F. contributed to the data analyses and the write up of the findings. M.N. reviewed the findings and commented on the manuscript. A.Jordan developed the idea for the research, reviewed the analyses and contributed greatly to the manuscript's write up. All authors discussed the results and commented on the manuscript.

## Supporting information


Data S1.

## References

[ejp70072-bib-0001] Alpers, G. W. , A. J. Winzelberg , C. Classen , et al. 2005. “Evaluation of Computerized Text Analysis in an Internet Breast Cancer Support Group.” Computers in Human Behavior 21, no. 2: 361–376. 10.1016/j.chb.2004.02.008.

[ejp70072-bib-0002] Bantum, E. O. C. , and J. E. Owen . 2009. “Evaluating the Validity of Computerized Content Analysis Programs for Identification of Emotional Expression in Cancer Narratives.” Psychological Assessment 21, no. 1: 79. 10.1037/a0014643. 19290768

[ejp70072-bib-0003] Beal, S. J. , and L. J. Crockett . 2010. “Adolescents' Occupational and Educational Aspirations and Expectations: Links to High School Activities and Adult Educational Attainment.” Developmental Psychology 46, no. 1: 258–265. 10.1037/a0017416. 20053022 PMC6379913

[ejp70072-bib-0004] Borucki, A. N. , and C. D. Greco . 2015. “An Update on Complex Regional Pain Syndromes in Children and Adolescents.” Current Opinion in Pediatrics 27, no. 4: 448–452. 10.1097/MOP.0000000000000250. 26087424

[ejp70072-bib-0005] Bujnowska, A. M. , C. Rodríguez , T. García , D. Areces , and N. V. Marsh . 2019. “Parenting and Future Anxiety: The Impact of Having a Child With Developmental Disabilities.” International Journal of Environmental Research and Public Health 16, no. 4: 668. 10.3390/ijerph16040668. 30823540 PMC6406654

[ejp70072-bib-0006] Campo, J. V. , J. Bridge , A. Lucas , et al. 2007. “Physical and Emotional Health of Mothers of Youth With Functional Abdominal Pain.” Archives of Pediatrics & Adolescent Medicine 161, no. 2: 131–137. 10.1001/archpedi.161.2.131. 17283297

[ejp70072-bib-0007] Clarke, V. , V. Braun , H. Frith , and N. Moller . 2019. “Editorial Introduction to the Special Issue: Using Story Completion Methods in Qualitative Research.” Qualitative Research in Psychology 16, no. 1: 1–20. 10.1080/14780887.2018.1536378.

[ejp70072-bib-0008] Cox, D. , J. L. McParland , and A. Jordan . 2022. “Parenting an Adolescent With Complex Regional Pain Syndrome: A Dyadic Qualitative Investigation of Resilience.” British Journal of Health Psychology 27, no. 1: 194–214. 10.1111/bjhp.12541. 34085746

[ejp70072-bib-0009] Cruz, N. , J. O'Reilly , B. S. Slomine , and C. F. Salorio . 2011. “Emotional and Neuropsychological Profiles of Children With Complex Regional Pain Syndrome Type‐I in an Inpatient Rehabilitation Setting.” Clinical Journal of Pain 27, no. 1: 27–34. 10.1097/ajp.0b013e3181f15d95. 20842016

[ejp70072-bib-0010] Eccleston, C. , G. Crombez , A. Scotford , J. Clinch , and H. Connell . 2004. “Adolescent Chronic Pain: Patterns and Predictors of Emotional Distress in Adolescents With Chronic Pain and Their Parents.” Pain 108, no. 3: 221–229. 10.1016/j.pain.2003.11.008. 15030941

[ejp70072-bib-0011] Forgeron, P. A. , J. Evans , P. J. McGrath , B. Stevens , and G. A. Finley . 2013. “Living With Difference: Exploring the Social Self of Adolescents With Chronic Pain.” Pain Research & Management 18, no. 6: e115. 10.1155/2013/120632. 24308027 PMC3917802

[ejp70072-bib-0012] Forgeron, P. A. , S. King , J. N. Stinson , P. J. McGrath , A. J. MacDonald , and C. T. Chambers . 2010. “Social Functioning and Peer Relationships in Children and Adolescents With Chronic Pain: A Systematic Review.” Pain Research and Management 15, no. 1: 27–41. 10.1155/2010/820407. 20195556 PMC2855293

[ejp70072-bib-0013] Francis, M. E. 1993. “Analysis of the Language and Process Dimensions Found in Personal Disclosure: The LIWC Approach [Doctoral Dissertation, Southern Methodist University].” Southern Methodist University ProQuest Dissertations Publishing.

[ejp70072-bib-0014] Frith, H. 2013. “Accounting for Orgasmic Absence: Exploring Heterosex Using the Story Completion Method.” Psychology & Sexuality 4, no. 3: 310–322. 10.1080/19419899.2012.760172.

[ejp70072-bib-0015] Gavin, H. 2005. “The Social Construction of the Child Sex Offender Explored by Narrative.” Qualitative Report 10, no. 3: 395–413. 10.46743/2160-3715/2005.1835.

[ejp70072-bib-0016] Hodges, L. , and B. Dibb . 2010. “Social Comparison Within Self‐Help Groups: Views of Parents of Children With Duchenne Muscular Dystrophy.” Journal of Health Psychology 15, no. 4: 483–492. 10.1177/1359105309355491. 20460405

[ejp70072-bib-0017] Huguet, A. , and J. Miró . 2008. “The Severity of Chronic Pediatric Pain: An Epidemiological Study.” Journal of Pain 9, no. 3: 226–236. 10.1016/j.jpain.2007.10.015. 18088558

[ejp70072-bib-0018] Irwin, S. , and S. Elley . 2013. “Parents' Hopes and Expectations for Their Children's Future Occupations.” Sociological Review 61, no. 1: 111–130. 10.1111/j.1467-954x.2012.02139.x.

[ejp70072-bib-0019] Jones, A. , L. Caes , C. Eccleston , M. Noel , T. Rugg , and A. Jordan . 2020. “Loss‐Adjusting: Young People's Constructions of a Future Living With Complex Regional Pain Syndrome.” Clinical Journal of Pain 36, no. 12: 932–939. 10.1097/ajp.0000000000000880. 32925189

[ejp70072-bib-0020] Jones, A. , L. Caes , J. Gauntlett‐Gilbert , and A. Jordan . 2023. “Defining Adolescence: A Call for Consistency in the Chronic Pain Literature.” Pediatric Pain Letter 24, no. 2: 9–13. http://ppl.childpain.org/issues/v25n2_2023/v25n2_jones.pdf.

[ejp70072-bib-0021] Jordan, A. L. , C. Eccleston , and M. Osborn . 2007. “Being a Parent of the Adolescent With Complex Chronic Pain: An Interpretative Phenomenological Analysis.” European Journal of Pain 11, no. 1: 49–56. 10.1016/j.ejpain.2005.12.012. 16458550

[ejp70072-bib-0022] Kahn, J. H. , R. M. Tobin , A. E. Massey , and J. A. Anderson . 2007. “Measuring Emotional Expression With the Linguistic Inquiry and Word Count.” American Journal of Psychology 120, no. 2: 263–286. 10.2307/20445398. 17650921

[ejp70072-bib-0023] Kim, S. , E. J. Rohn , and A. L. Kratz . 2021. “Linguistic Indicators of Chronic Pain Acceptance in Individuals With Spinal Cord Injury.” Rehabilitation Psychology 66, no. 4: 520–531. 10.1037/rep0000399. 34516167

[ejp70072-bib-0024] Klein, K. , and A. Boals . 2010. “Coherence and Narrative Structure in Personal Accounts of Stressful Experiences.” Journal of Social and Clinical Psychology 29, no. 3: 256–280. 10.1521/jscp.2010.29.3.256.

[ejp70072-bib-0025] Koning, I. M. , R. J. van den Eijnden , T. Glatz , and W. A. Vollebergh . 2013. “Don't Worry! Parental Worries, Alcohol‐Specific Parenting and Adolescents' Drinking.” Cognitive Therapy and Research 37: 1079–1088. 10.1007/s10608-013-9545-0.

[ejp70072-bib-0026] Langer, S. L. , J. M. Romano , Q. Liu , R. L. Levy , H. Nielson , and J. D. Brown . 2016. “Pain Catastrophizing Predicts Verbal Expression Among Children With Chronic Pain and Their Mothers.” Health Psychology Open 3, no. 1. 10.1177/2055102916632667. PMC5193312 28070387

[ejp70072-bib-0027] Le, A. , B. R. Dick , J. Spiers , K. Reid , and S. D. Scott . 2019. “Parents' Experiences With Pediatric Chronic Pain.” Canadian Journal of Pain 3, no. 1: 20–32. 10.1080/24740527.2019.1577679. 35005391 PMC8730635

[ejp70072-bib-0028] Logan, D. E. , S. E. Williams , V. P. Carullo , R. L. Claar , S. Bruehl , and C. B. Berde . 2013. “Children and Adolescents With Complex Regional Pain Syndrome: More Psychologically Distressed Than Other Children in Pain?” Pain Research and Management 18, no. 2: 87–93. 10.1155/2013/964352. 23662291 PMC3718058

[ejp70072-bib-0029] McDade, T. W. , L. Chyu , G. J. Duncan , L. T. Hoyt , L. D. Doane , and E. K. Adam . 2011. “Adolescents' Expectations for the Future Predict Health Behaviors in Early Adulthood.” Social Science & Medicine 73, no. 3: 391–398. 10.1016/j.socscimed.2011.06.005. 21764487 PMC3148854

[ejp70072-bib-0030] Mendes, T. P. , C. A. Crespo , and J. K. Austin . 2017. “The Psychological Costs of Comparisons: Parents' Social Comparison Moderates the Links Between Family Management of Epilepsy and Children's Outcomes.” Epilepsy & Behavior 75: 42–49. 10.1016/j.yebeh.2017.07.017. 28826008

[ejp70072-bib-0031] Nilsson, I. M. , M. Drangsholt , and T. List . 2009. “Impact of Temporomandibular Disorder Pain in Adolescents: Differences by Age and Gender.” Journal of Orofacial Pain 23, no. 2: 115–122. 19492536

[ejp70072-bib-0032] Nimbley, E. , L. Caes , A. Jones , E. Fisher , M. Noel , and A. Jordan . 2021. “A Linguistic Analysis of Future Narratives in Adolescents With Complex Regional Pain Syndrome and Their Pain‐Free Peers.” European Journal of Pain 25, no. 3: 693–703. 10.1002/ejp.1704. 33259699

[ejp70072-bib-0033] O'Donnell, A. W. , G. Redmond , J. Arciuli , et al. 2022. “The Association Between Parental Educational Expectations and School Functioning Among Young People With Disabilities: A Longitudinal Investigation.” Exceptional Children 89, no. 1: 60–78. 10.1177/00144029221087392.

[ejp70072-bib-0034] Parsons, R. D. , J. L. McParland , S. L. Halligan , L. Goubert , and A. Jordan . 2022. “Flourishing Among Adolescents Living With Chronic Pain and Their Parents: A Scoping Review.” Paediatric and Neonatal Pain 4, no. 4: 158–168. 10.1002/pne2.12088. 36618512 PMC9798043

[ejp70072-bib-0035] Pasupathi, M. 2007. “Telling and the Remembered Self: Linguistic Differences in Memories for Previously Disclosed and Previously Undisclosed Events.” Memory 15, no. 3: 258–270. 10.1080/09658210701256456. 17454663

[ejp70072-bib-0036] Peeters, M. A. , S. R. Hilberink , and A. van Staa . 2014. “The Road to Independence: Lived Experiences of Youth With Chronic Conditions and Their Parents Compared.” Journal of Pediatric Rehabilitation Medicine 7, no. 1: 33–42. 10.3233/prm-140272. 24919936

[ejp70072-bib-0037] Pennebaker, J. W. , R. J. Booth , R. L. Boyd , and M. E. Francis . 2015. Linguistic Inquiry and Word Count: LIWC2015 Operator's Manual. Pennebaker Conglomerates.

[ejp70072-bib-0038] Pennebaker, J. W. , R. L. Boyd , K. Jordan , and K. Blackburn . 2015. “The Development and Psychometric Properties of LIWC2015.” https://repositories.lib.utexas.edu/handle/2152/31333.

[ejp70072-bib-0039] Pennebaker, J. W. , and M. E. Francis . 1996. “Cognitive, Emotional, and Language Processes in Disclosure.” Cognition & Emotion 10, no. 6: 601–626. 10.1080/026999396380079.

[ejp70072-bib-0040] Pennebaker, J. W. , M. E. Francis , and R. J. Booth . 2001. Linguistic Inquiry and Word Count: LIWC. Lawrence Erlbaum Associates.

[ejp70072-bib-0041] Pilny, A. , K. McAninch , A. Slone , and K. Moore . 2019. “Using Supervised Machine Learning in Automated Content Analysis: An Example Using Relational Uncertainty.” Communication Methods and Measures 13, no. 4: 287–304. 10.1080/19312458.2019.1650166.

[ejp70072-bib-0042] Pinquart, M. , and M. Ebeling . 2020. “Parental Educational Expectations and Academic Achievement in Children and Adolescents—A Meta‐Analysis.” Educational Psychology Review 32: 463–480. 10.1007/s10648-019-09506-z.

[ejp70072-bib-0043] Prego‐Domínguez, J. , Z. Khazaeipour , N. Mallah , and B. Takkouche . 2021. “Socioeconomic Status and Occurrence of Chronic Pain: A Meta‐Analysis.” Rheumatology 60, no. 3: 1091–1105. 10.1093/rheumatology/keaa758. 33276382

[ejp70072-bib-0044] Qualtrics . 2019. “Three Powerful Suites for Optimizing Experiences Across Your Business.” https://www.qualtrics.com.

[ejp70072-bib-0045] Sawyer, S. M. , P. S. Azzopardi , D. Wickremarathne , and G. C. Patton . 2018. “The Age of Adolescence.” Lancet Child & Adolescent Health 2, no. 3: 223–228. 10.1016/s2352-4642(18)30022-1. 30169257

[ejp70072-bib-0046] Schmid, K. L. , and S. J. Lopez . 2011. “Positive Pathways to Adulthood: The Role of Hope in Adolescents' Constructions of Their Futures.” Advances in Child Development and Behavior 41: 69–88. 10.1016/B978-0-12-386492-5.00004-X. 23259189

[ejp70072-bib-0047] Soltani, S. , D. C. Kopala‐Sibley , and M. Noel . 2019. “The Co‐Occurrence of Pediatric Chronic Pain and Depression.” Clinical Journal of Pain 35, no. 7: 633–643. 10.1097/ajp.0000000000000723. 31094934

[ejp70072-bib-0048] Tausczik, Y. R. , and J. W. Pennebaker . 2010. “The Psychological Meaning of Words: LIWC and Computerized Text Analysis Methods.” Journal of Language and Social Psychology 29, no. 1: 24–51. 10.1177/0261927X09351676.

[ejp70072-bib-0049] Van Alboom, M. , T. Elmer , K. Boersma , et al. 2022. “Social Integration of Adolescents With Chronic Pain: A Social Network Analysis.” Pain 163, no. 11: 2232–2244. 10.1097/j.pain.0000000000002623. 35439797

[ejp70072-bib-0050] Vetter, T. R. , C. L. Bridgewater , and G. McGwin . 2012. “An Observational Study of Patient Versus Parental Perceptions of Health‐Related Quality of Life in Children and Adolescents With a Chronic Pain Condition: Who Should the Clinician Believe?” Health and Quality of Life Outcomes 10, no. 1: 1–12. 10.1186/1477-7525-10-85. 22824550 PMC3478968

[ejp70072-bib-0051] Wakefield, E. O. , W. T. Zempsky , R. M. Puhl , and M. D. Litt . 2018. “Conceptualizing Pain‐Related Stigma in Adolescent Chronic Pain: A Literature Review and Preliminary Focus Group Findings.” Pain Reports 3, no. 1: e679. 10.1097/pr9.0000000000000679. 30324171 PMC6172824

[ejp70072-bib-0052] Weissmann, R. , and Y. Uziel . 2016. “Pediatric Complex Regional Pain Syndrome: A Review.” Pediatric Rheumatology 14, no. 1: 1–10. 10.1186/s12969-016-0090-8. 27130211 PMC4850724

