# Supplementary material for: A Linguistic Analysis of Future Perceptions of Parents of Adolescents With Complex Regional Pain Syndrome and Parents of Pain‐Free Peers
Source: Eur J Pain. 2025 Jun 27;29(6):e70072. doi: 10.1002/ejp.70072 (PMC12203762; doi:10.1002/ejp.70072)
Supplement: Supplementary file 1 — Data S1. [file EJP-29-0-s001.docx]

| Participant ID | Word/phrase (relevant to categories included) in bolded text | Evidence of negation | Relevant LIWC subcategory |
| --- | --- | --- | --- |
| HP2 | **Happily** living  **Possibly** somewhere  Working with her ex university **friends**  **Probably** also have had a **child**  Partner may be the biological **mother**  Early years post uni as **great** **fun**  Very **hard** work | None  None  None  None  None  None  None | Positive emotion  Tentativeness  Friends  Tentativeness, Family  Family  Positive emotion (two occurrences)  Negative emotion |
| HP6 | **Good** qualifications  It is **hard** work  I **worry** that I will not earn enough  Someone in the **family** **dies**  **Enjoy** myself in other ways  Close relationship with my **mum**  I get **angry** at times  Who **died** when I was 13  Life is **unfair** | None  None  None  None  None  None  None  None  None | Positive emotion  Negative emotion  Anxiety  Family, Negative emotion  Positive emotion  Family  Angry  Negative emotion  Anger, Negative emotion,Sadness |
| HP13 | **Happy** life  Cause them **anxiety** and are **fearful**  Having **children**  Future is **undecided** | None  None  None  None | Positive emotion  Anxiety, Negative emotion  Family word  Tentative, Discrepancy |
| HP17 | Living with his own **family** or **friends**  **May** or **may** not have a **family** of his own  With his little **brother**  Be able to manage his **anxiety** | None  Yes, but [may or may not] negate each other’s effect  None  None | Family, Friends  Tentativeness (two occurrences), Family  Family  Anxiety |
| HP23 | Will be of **low** mood  Will not find life very **fulfilling**  he **may**  he **may**  he **may**  Will not have fallen in **love** | None  Yes [not]  None  None  None  Yes [not] | Negative emotion  Positive emotion  Tentativeness  Tentativeness  Tentativeness  Positive emotion |
| HP28 | Found a career she **enjoys**  **Wellbeing** and achievement  **Happy**, **loving** relationship  Treat her **badly**  **Kind** and **happy**  **Children** | None  None  None  None  None  None | Positive emotion  Positive emotion  Positive emotion (two occurrences)  Negative emotion  Positive emotion (two occurrences)  Family |
| HP32 | **Children**  **Nephew**, **niece**, **brothers**, **stepdad**, **grandad**  **Funny** | None  None  None | Family  Family (five occurrences)  Positive emotion |
| HP36 | She **may** have changed jobs  **Confidence**  She **may** have been travelling  She **may possibly** have entered  She **may** have met a partner  **Enjoying** her independence  **Suffering** a little from **mental** health **issues**  **But** I think  **Positive** mindset | None  None  None  None  None  None  None  None  None | Tentativeness  Positive emotion  Tentativeness  Tentativeness (two occurrences)  Tentativeness  Positive emotion  Negative emotion, Insight, Negative emotion  Differentiation  Positive emotion |
| HP45 | **But** pulled out after one year  **Because** I had met my partner  Going to have a **baby**  **Son** was born  **But** his arrival led to big changes  A new **father**  **Son’s** birth  **Son**  **But** am not sure how I would be able to cope  **Worried** about finding a balance  **Because** she feels like she  **But fear** that I might be cooking forever | None  None  None  None  None  None  None  None  None  None  None  None | Differentiation  Causation  Family  Family  Differentiation  Family  Family  Family  Differentiation  Anxiety, Negative emotion  Causation  Differentiation, Anxiety, Negative emotion |
| HP50 | **Although** it was a bit **scary**  A newfound **confidence**  An unexpected **passion**  Still **loves** music  Living in Glasgow with **friends**  **But** for now is **happy** to be where she is  She is **passionate** about | None  None  None  None  None  None  None | Differentiation, Anxiety, Negative emotion  Positive emotion  Positive emotion  Positive emotion  Friends  Differentiation, Positive emotion  Positive emotion |
| CP1 | **Trauma**  **Abuse** of **trust**  His **painful** experience  **Confidence** and **trust** issues  What he wants and **enjoys**  He **may** be a scientist  He’ll find it very **hard** | None  Yes [Abuse]  None  Yes [issues]  None  None  None | Negative emotion  Negative emotion, Positive emotion  Negative emotion  Positive emotion, Positive emotion  Positive emotion  Tentativeness  Negative emotion |
| CP2 | She would **love** to attend  Social **anxiety**  She would **flourish** in this  **But** she has **never** been **pain** free  She will have a small group of **friends**  **But** the reality is  She is not very **good** at maintaining **friendships**  **But** I feel that she will be **happy** in that world | None  None  None  None  None  None  Yes [Not]  None | Positive emotion  Negative emotion, Anxiety  Positive emotion  Differentiation, Certainty, Negative emotion  Friends  Differentiation  Positive emotion, Friends  Differentiation, Positive emotion |
| CP6 | She has a few **good friends**  Doesn’t **trust** very many people  In lots of **pain**  **Enjoys** the cinema  She will be **proud**  **Thought** she needed | None  Yes [doesn’t]  None  None  None  None | Positive emotion, friends  Positive emotion  Negative emotion  Positive emotion  Positive emotion  Insight |
| CP13 | She is more **positive**  **Enjoys** time with her **friends**  **Confidently**  Holiday with her **sisters**  She is **happier**  Manages her **pain**  Her **confidence** is getting back  Without asking a **family** member  Self **confidence** | None  None  None  None  None  None  None  Yes [without]  None | Positive emotion  Positive emotion, Friends  Positive emotion  Family  Positive emotion  Negative emotion  Positive emotion  Family  Positive emotion |
| CP17 | **Favourite**  Will have met new **friends**  Will still be **friends** with  **Never** be shy  Continue to do **good** deeds  **Marriage** and **family**  Fight through her **pain** | None  None  None  None  None  None  None | Positive emotion  Friends  Friends  Certainty  Positive emotion  Family, Family  Negative emotion |
| CP23 | **Hard** to plan  It is so **hard**  Make a **family**  Having **children** | None  None  None  None | Negative emotion  Negative emotion  Family  Family |
| CP28 | I am **hopeful**  Which he **dislikes**  Not **worry** about looking  He **loves** his job  **Fulfilling** relationship and **happy** | None  None  Yes [not]  None  None | Positive emotion  Negative emotion  Anxiety, Negative emotion  Positive emotion  Positive emotion (two occurrences) |
| CP32 | Managing her **pain** well  In a profoundly **positive** way  Feel **good**  Meets **great friends**  she’ll **learn** how to  Without **dwelling** on her **pain**  who takes her **pain**  the **pain** that she has  will **affect** the way she | None  None  None  None  Yes [without]  None  None  None  None | Negative emotion  Positive emotion  Positive emotion  Positive emotion, Friends  Insight  Insight, Negative emotion  Negative emotion  Negative emotion  Causation |
| CP38 | **Like** to be a music producer  **Hard** due to her **pain** and fatigue  She will **struggle**  Her **friends** do  It then **causes** a flare up  **Pain** can become extreme  Will **struggle** to live alone  Sees her **friends** able to work  A **good** social life  **Struggles** physically | None  None  None  None  None  None  None  None  None  None | Positive emotion  Negative emotion (two occurrences)  Negative emotion  Friends  Causation  Negative emotion  Negative emotion  Friends  Positive emotion  Negative emotion |
| CP45 | **But** got my degree  Best **friend**  it was **difficult**  **but** I can work from home  When I’m feeling **good**  **Like** to be in an office around people  **But** I’ve found a job I **like**  **Good** support system of **friends** and **family** | None  None  None  None  None  None  None  None | Differentiation  Friends  Negative emotion  Differentiation  Positive emotion  Positive emotion  Differentiation, Positive emotion  Positive emotion, Friends, Family |

Supplementary Table 1: Examples of 20 randomly sampled responses across both groups (n=10 responses from parents of adolescents with CRPS and n=10 responses from parents of pain-free adolescents) to examine presence of negations in relation to the relevant LIWC categories.
